# Inside the European Plant Viroid Scenario: Continental Distribution, Host Range, and Genetic Features of the Main Viroid Populations

**DOI:** 10.3390/v18030325

**Published:** 2026-03-05

**Authors:** Athos Pedrelli, Marzia Vergine, Luigi De Bellis, Andrea Luvisi

**Affiliations:** 1 Department of Biological and Environmental Sciences and Technologies, University of Salento, 73100 Lecce, Italy; marzia.vergine@unisalento.it (M.V.); luigi.debellis@unisalento.it (L.D.B.); andrea.luvisi@unisalento.it (A.L.); 2 National Biodiversity Future Center, 90133 Palermo, Italy

**Keywords:** use classification, growth form, HSVd, CEVd, PSTVd, PLMVd, CSVd, sequence analyses

## Abstract

Viroids are a serious threat to plant health due to their broad host range, high infectivity, and latent infections. Europe’s heterogeneous climate, ecology, and agriculture make it a key setting for viroid research. Despite numerous country- and host-specific reports, a continental synthesis has been lacking. In this study, we systematically collected all available official records of plant viroids in Europe from 1972 to 2025. A total of 255 documents were analyzed, encompassing 35 countries of the European continent and 118 host plant species, classified by host use (cultivated, ornamental, wild) and growth habit. Nucleotide sequences of the most common European viroids were retrieved from the NCBI database to assess genetic diversity and recombination. Europe hosts 32 of the 45 recognized viroid species worldwide (~71%), representing all eight genera. Southern Europe emerged as the main hotspot (~70% of reports), largely associated with Mediterranean climates and intensive cultivation of woody crops. Cultivated plants were the dominant hosts across all regions, while ornamentals were particularly important in Northern and Western Europe. Population genetic analyses revealed heterogeneous patterns, quasispecies dynamics, and recombination, shaped by host and geography. This is the first integrated overview of viroids across Europe, highlighting the importance of surveillance, sequencing, and genomic research.

## 1. Introduction

The European continent, with approximately 10.18 million square kilometers, exhibits extraordinary geographical, climatic, and ecological diversity, bounded by the Atlantic Ocean to the west, the Arctic Ocean to the north, and the Mediterranean, Black, and Caspian Seas to the south, with the Ural Mountains and River delineating its eastern frontier with Asia [1,2]. Europe includes more than 50 nations, including geographically European sovereign states, transcontinental countries with partial European territories such as Russia, Türkiye, Kazakhstan, Azerbaijan, and Georgia. Its varied landscape ranges from northern tundra and glacial fjords to alpine chains such as the Alps and the Carpathians, fertile central plains, and Mediterranean coastal regions. Climatic zones span subarctic, temperate, and Mediterranean bands, contributing to the continent’s agroecological heterogeneity [3].

In this complex scenario, further challenged by climate change, cultivated plants for food, feed, or ornamental purposes, as well as wild species in landscapes, constantly face the threat of plant pathogens, which can have devastating effects on production and, consequently, a strong socio-economic impact on the well-being of countries [4,5,6,7].

Viroids rank among the most concerning plant pathogens and are recognized as the smallest infectious agents known. These obligate parasites consist solely of single-stranded, circular RNA molecules measuring between 240 and 440 nucleotides in length, and they lack a protective coat protein [8]. Despite their structural simplicity, viroids can cause severe plant diseases, a threat further heightened by their ability to spread through various ways [9,10]. Vegetative propagation, grafting, and mechanical transmission (via contaminated hands, pruning, and harvesting tools) are the most prevalent modes at both global and local scales [11,12]. However, seed and pollen transmission can occur for some viroids [13]. In addition, some biological vectors, such as insects, parasitic plants, and goats, are known to transmit viroids [14,15]. Notably, recent studies have demonstrated that viroids can replicate within and be transmitted by phytopathogenic ascomycete fungi under laboratory conditions, suggesting that fungi may also act as natural vectors [16].

Although symptoms associated with viroids had been documented as early as the 1920s and were initially attributed to a putative viral agent [17,18], the first identification was achieved in 1971 by Diener [19,20], who recognized *Pospiviroid fusituberis* (PSTVd) as the etiological agent of potato spindle tuber disease in the United States of America. Since this discovery, it has become evident that viroid infections can induce a wide range of symptoms, whose expression varies with the host species and environmental conditions, particularly favored by high temperatures (25 °C to 35 °C) and light [21,22,23,24]. Typical symptoms include stunting, chlorosis, leaf deformation, fruit malformation, and bark necrosis. In severe cases, they may lead to plant death. However, some viroids remain asymptomatic, hindering detection and management [18,25]. Interestingly, symptoms induced by viroids seem to be closely linked to their ability to interfere with host gene regulation through RNA silencing mechanisms [26].

Therefore, they represent a considerable threat to agriculture due to their wide host range, spanning from woody to herbaceous species, and affecting major crops such as grapevine, stone fruits, pome fruits, horticultural crops, and ornamental plants [27,28,29,30]. As a result, yield losses associated with these pathogens can exceed 20% and, in some cases, compromise the entire harvest [31,32,33], leading to reduced product quality and marketability, resulting in extensive economic damage [34,35]. Moreover, additional economic burdens arise from increased field management costs for viroid diseases, as well as from the need for strict certification procedures for commercial plant materials to prevent their spread into unaffected areas [36]. For these reasons, some viroids are classified as quarantine pests (e.g., *Cocadviroid cadangi*, CCCVd) or regulated non-quarantine pests (RNQP) that must be excluded from propagation material (e.g., *Apscaviroid cicatricimali*, ASSVd; *Pospiviroid exocortiscitri*, CEVd; *Hostuviroid impedihumuli*, HSVd; *Pospiviroid fusituberis*, PSTVd) by the European Union [37].

Current taxonomy classifies viroids into two primary families based on distinct differences in genome structure, replication sites and mechanisms, and the presence or absence of ribozyme sequences [38,39]. To date, 45 viroid species have been identified [40]. Members of the family Pospiviroidae are replicated in the nucleus via an asymmetric rolling-circle mechanism and include the genera *Apscaviroid* (19 species), *Pospiviroid* (10), *Coleviroid* (5), *Cocadviroid* (4), and *Hostuviroid* (2). They typically adopt a rod-shaped secondary structure, functionally divided into five domains, including a conserved central region. In contrast, viroids of the family Avsunviroidae are replicated in the chloroplast via a symmetric rolling-circle mechanism. This family includes the genera *Pelamoviroid* (3 species), *Avsunviroid* (1 species), and *Elaviroid* (1 species). Their structures may be rod-shaped, branched, or semi-branched and are characterized by the presence of self-cleaving hammerhead ribozyme sequences [41,42,43]. Unlike viruses, viroids do not encode any proteins and rely entirely on the host’s cellular machinery for replication and movement [9].

Detecting viroids through bioassays has proven challenging, particularly when mixed infections are involved, as different viroid variants can mask or reduce symptom expression. Moreover, viroids exhibit high genetic variability, and even a single nucleotide change can significantly affect symptom manifestation. Complicating matters further, different viroid species, such as those within the *Pospiviroid* genus, can produce similar symptoms on the same indicator host plant [24]. Additionally, because they are non-coding and lack protein expression, viroids do not produce structural or non-structural proteins capable of triggering immune responses or serving as antigenic markers. As a result, only the recent advances in detection technologies have unveiled novel insights into viroids, highlighting their diffusion on a global and regional scale, even in regions previously considered unaffected, and identifying both new and previously reported viroids in different areas [44,45,46,47]. To the best of our knowledge, only a few studies have realized a systematic synthesis of all or partial available data across European countries [48,49]. To bridge this gap and summarize the latest research on this topic, a comprehensive analysis was conducted with the aim of (i) describing the evolution of European research across decades, presenting data on viroid research in Europe from the perspectives of (ii) country, (iii) host, (iv) viroid, and (v) providing insights into the most widespread reported viroid in Europe, including its symptoms and population studies.

## 2. Data Recovery and Bioinformatics Analyses

A database was created in July 2025 examining (i) peer-reviewed articles, (ii) institutional documents, (iii) conference proceedings, and (iv) an international database concerning all available data about viorids diffusion in European countries. The data were searched in the Web of Science (Thompson-ISI, Philadelphia, PA, USA), Google Scholar (Google, Mountain View, CA USA), Scopus (Elsevier, Amsterdam, Netherlands), and European and Mediterranean Plant Protection Organization (EPPO; https://gd.eppo.int/, accessed on 30 July 2025) database using multiple combinations of “viroid”, “diagnosis”, “diffusion”, “epidemiology”, “first report”, “host”, “identification”, “report”, and the single name of every 51 European countries (Supplementary Table S1). The reference lists of all articles identified through the literature search were cross-checked to include any additional relevant sources, resulting in a total of 255 documents. The collected data were examined to extract the following information: (i) viroid identified, (ii) year of report, (iii) host species in which the viroid was detected, and (iv) country of report, regardless of whether the record represented the first detection of the viroid and/or the host. To better analyze data, European countries showing viroids reports were enclosed in geographical areas as follows: Central Europe (CE), Eastern Europe (EE), Northern Europe (NE), Southern Europe (SE) and Western Europe (WE). Moreover, each host species record was classified by use (cultivated, ornamental, or wild plants) and growth form (woody, shrub, or herbaceous plants). Abbreviations of single species, i.e., sp., or multiple species, i.e., spp., associated with a genus were maintained as recovered by documents. All the recovered data were reported in an ad hoc database (Supplementary Material S1).

Finally, the main European viroids identified have undergone a focus on symptoms and genetic features based on nucleotide sequences reported in Europe. Nucleotide sequences of these viroids were retrieved from the NCBI database (https://www.ncbi.nlm.nih.gov/, accessed on 30 July 2025), considering only those in which the “country” field was directly reported, and downloaded for analysis. The sequences were organized into different viroid datasets, and each sequence was named as follows: accession number, host (if present), and country/countries. Sequences were aligned using BioEdit [50] and manually checked to remove ambiguous sequences in the populations (Supplementary Table S2). The recombinant events in the nucleotide sequences were evaluated using the RDP4 program (v. 4.39) with 3Seq, Bootscan, Chimaera, GENECONV, MaxChi, RDP, and SiScan algorithms [51]. They were accepted only if identified by at least four methods (*p* < 0.05) and a significant *p*-value displayed is due to multiple comparison (MC) correction (*p* < 0.05). Nucleotide diversity (π), haplotype numbers, haplotype diversity (Hd), and Tajima’s neutrality test (D) were calculated for each viroid population to evaluate genetic variability and potential signatures of selection. To assess the contribution of taxonomic and geographic factors to genomic variation, an Analysis of Molecular Variance (AMOVA) was conducted, considering three grouping factors: country, genus, and country × genus. To ensure comparability across groups and avoid statistical bias, groups with fewer than three sequences were excluded because very small sample sizes can artificially inflate or distort variance estimates and reduce the robustness of the analysis. From each remaining group, up to ten sequences were randomly selected (or all sequences were included if fewer than ten were available). Finally, the significance (*p* < 0.05) of group differentiation was evaluated using permutation tests (1000 permutations), and Phi-statistics (Φ_ST_) were used to quantify the proportion of genetic variance explained by group structure. These analyses were performed using the ‘*pegas*’ package (Population and Evolutionary Genetics Analysis System) in R Studio (Version 2026.01.0+392) [52,53].

## 3. Distribution and Epidemiology of Viroids in Europe

### 3.1. Temporal and Geographic Trends

**Figure 1:**
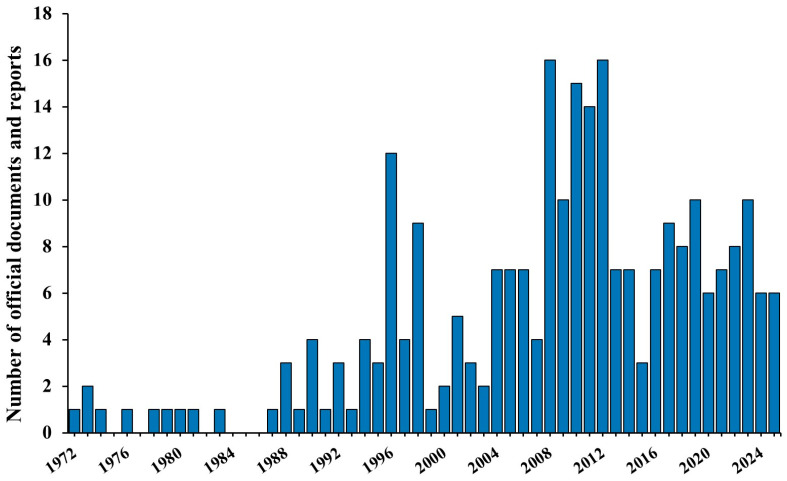
Annual number of peer-reviewed articles and official documents (1972–2025) on viroids reporting new species, new hosts, or new country records in Europe, as retrieved in this study. The introduction of molecular techniques in the late 1980s led to an increase in viroid research, with a marked rise in the late 1990s driven by advances in classification efforts carried out by the International Committee on Taxonomy of Viruses (ICTV), and again in the late 2000s with the adoption of high-throughput sequencing (HTS), which enabled the detection of all RNA pathogens present in infected plants. For the years not reported, no documents were available.

Viroid research in Europe was closely linked to the evolution of molecular tools, with the late 1990s and 2010s representing the periods with the highest number of reports. This increase also reflects the implementation of surveillance programs in several European countries after late-1990s findings revealed that various ornamental plants harbored multiple pospiviroids. This progression is clear in the collected documents, which cover a 54-year period (1972–2025; Figure 1).

Although serological techniques commonly employed for virus detection were available from the 1970s, they were ineffective for identifying viroids [54]. As a result, from their discovery until the late 1980s, only a few reports appeared due to the lack of sensitive and specific diagnostic tools capable of detecting their presence in host plants [55,56]. The introduction of new molecular techniques in 1987 led to the development of more precise, reliable, and rapid tools, unlocking the ability not only to detect viroids but also to study their structure, thereby fueling renewed interest [57,58]. One of the early techniques, polyacrylamide gel electrophoresis (PAGE), was among the first significant breakthroughs [59,60,61]. Over time, nucleic acid-based approaches such as hybridization with labeled probes and reverse transcription polymerase chain reaction (RT-PCR) became standard tools [62,63,64,65]. In the 1990s, viroids were classified for the first time, and studies describing their structure and distribution proliferated, particularly in the latter part of the decade [41]. More recently, reverse transcription loop-mediated isothermal amplification (RT-LAMP) and next-generation sequencing (NGS) have enabled faster and more sensitive viroid detection [66,67,68,69]. As a result, a second surge of epidemiological work followed in the late 2000s, driven by high-throughput sequencing (HTS), which revealed all RNA pathogens in infected plants and uncovered previously unreported viroids [70]. From 2013 onward, new viroid publications stabilized at around seven per year, while research increasingly focused on the mechanisms of plant-viroid interactions [71,72,73]. Together, this expanding body of work has dramatically deepened our understanding of viroid biology, especially in epidemiological studies, highlighting their substantial presence across Europe.

These methodological advances not only accelerated viroid detection but also reshaped the geographic landscape of viroid reports. As diagnostic capacity improved, viroids were progressively identified in an increasing number of countries, revealing clear temporal and spatial trends in their distribution.

**Figure 2:**
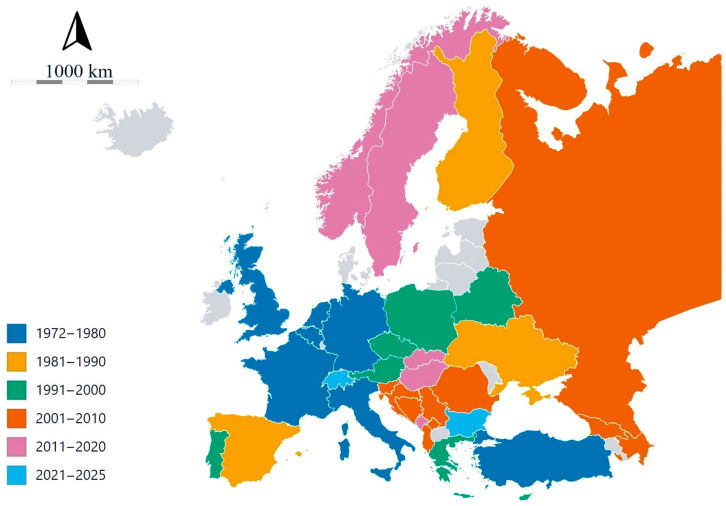
Timeline of viroid reports across Europe. Different colors highlight the decades in which the first report was made for each country. The chronological pattern reflects both the heterogeneous intensity of surveillance among countries and the progressive improvement in diagnostic capacity driven by the introduction of new molecular techniques and the expansion of intra-European trade and plant material exchange. Countries without reports are shown in gray.

Currently, viroids have been reported in 35 of 51 European countries, encompassing geographically European sovereign states and transcontinental nations with European territories (Figure 2).

The earliest European reports appeared in Belgium (1972), soon followed by Germany (1973), the United Kingdom (1973), the Netherlands (1974), France (1976), Italy (1978) and Türkiye (1979). During the 1980s and 1990s, viroid detections spread steadily north and east, with records from Finland (1981), Spain (1987), Ukraine (1990), Cyprus (1992), Czechia (1994), Portugal (1995), Poland (1994), Austria (1996) and finally Belarus and Greece (1998). In the 2000s through the early 2020s, reports became more geographically widespread, encompassing Albania (2004), Bosnia and Herzegovina (2005), Kosovo and Romania (2006), Croatia and Serbia (2008), and Azerbaijan, Georgia, and Russia (European part; 2009). Subsequent detections were recorded in Slovenia (2010), Norway (2011), Montenegro (2012), Slovakia (2016), Sweden (2017), and Hungary (2018), with the most recent records from Switzerland (2023), Malta (2024), and Bulgaria (2025). No detections were documented in Andorra, Armenia, Denmark, Estonia, Iceland, Ireland, Kazakhstan (European part), Latvia, Liechtenstein, Lithuania, Luxembourg, Moldova, Monaco, North Macedonia, San Marino, and Vatican City. Based on these data, the geographical regions were defined as follows: NE including Finland, Norway, Sweden, and United Kingdom; CE with Austria, Czechia, Germany, Hungary, Poland, Slovakia, and Switzerland; EE including Azerbaijan, Belarus, Georgia, Romania, Russia (European part), and Ukraine; SE with Albania, Bosnia and Herzegovina, Bulgaria, Croatia, Cyprus, Greece, Italy, Kosovo, Malta, Montenegro, Portugal, Serbia, Slovenia, Spain, and Türkiye; WE including Belgium, France, and Netherlands.

The chronological pattern reflects both the heterogeneous intensity of surveillance among countries and the progressive improvement in diagnostic capacity and awareness of viroid threats, driven by the introduction of new molecular techniques and the expansion of intra-European trade and plant material exchange [24,74]. Indeed, countries with well-developed research infrastructures and subject to intense import activity of plant materials (e.g., Italy, France, and Germany) detected viroids earlier, whereas others may have experienced delayed or underreported occurrences, likely due to limited research capacity or low plant import.

### 3.2. Geographical Distribution and Host Types

**Figure 3:**
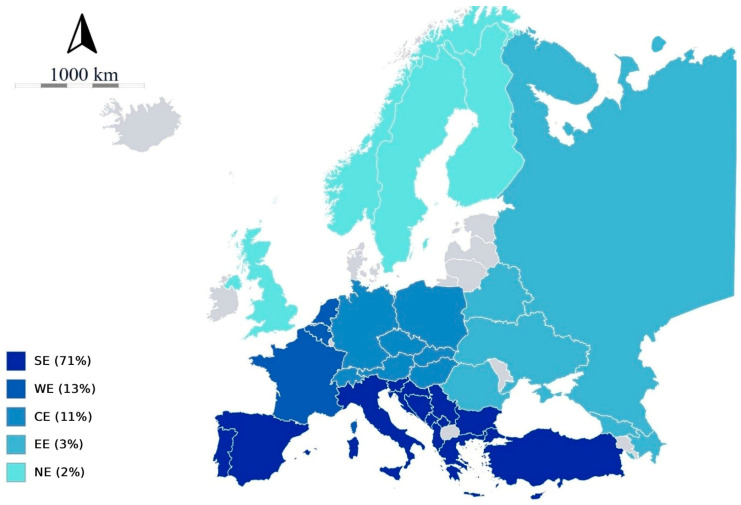
Distribution and percentage (%) of reports of new or established viroids across the five European macroareas, illustrated in color-coded maps. Southern Europe shows the highest number of viroid occurrences among all regions. CE = Central Europe; EE = Eastern Europe; NE = Northern Europe; SE = Southern Europe; WE = Western Europe.

Southern Europe emerged as the central hub for viroids, accounting for approximately 70% of the reports of new or established viroids in investigated host plants (Figure 3).

This was followed by WE at around 13%, CE at around 11%, and EE and NE at approximately 3% and 2%, respectively. Italy, France, Czechia, Russia, and Finland, respectively, are the countries with major reports within the geographical regions. However, only Italy (first) and France (fifth) ranked among the European top five, with the remaining positions occupied by Greece (second), Türkiye (third), and Spain (fourth; Supplementary Table S3). Indeed, the Mediterranean countries above reported collectively accounted for approximately 56% of all reports at the European level and for 79% of SE reports, showing their prominence in viroid reports and studies.

The result is not surprising, given the Mediterranean basin’s central role as a hotspot for viroids. This is driven by its warm climate, often aligning with the optimal thermal range for viroid activity (25 °C to 35 °C), and by socio-cultural practices that have facilitated their transmission across diverse host plant species [21,75,76]. Those factors enhance the replication, transmission, and persistence of viroids in susceptible host plants, contributing significantly to their epidemiological dynamics in the region [77]. Consequently, the increasing temperatures due to climate change represent a growing challenge for the European continent and raise awareness of their potential to increase viroid presence and facilitate their spread into regions where they were previously limited or absent [5]. This expansion could be further accelerated by cultivating new crops that serve as viroid hosts, especially in areas previously considered unsuitable for such agriculture due to climatic constraints [78]. In a forward-looking perspective, these variations highlight the need for strengthened surveillance strategies, including the systematic monitoring of emerging putative viroid hosts, particularly in northern latitudes. This requires integrating established methodologies (i.e., ELISA, PCR) with innovative approaches such as predictive modeling and early-detection tools (i.e., Artificial Intelligence models, satellites), combined into multi-source early-warning networks capable of supporting timely phytosanitary interventions, similar to what is already being developed for other plant pathogens within the European STELLA Pest Surveillance System (PSS) project [79].

Fifty-three genera are recognized as viroid hosts, involving around 117 species (Table 1).

Cultivated plants represent the majority (about 59%) of infected species, followed by ornamentals (32%) and wild plants (9%). Around 68 cultivated species are reported as hosts, including woody (52 species), herbaceous (10), and shrub (6) species. The most frequently reported are *Vitis vinifera* (L.), *Prunus persica* (L.) Batsch, *Prunus armeniaca* (L.), *Solanum lycopersicum* (L.), and *Citrus × limon*. Ornamentals include about 39 species, predominantly herbaceous (22), followed by shrubs (14) and woody plants (3). The most common ornamental hosts are *Solanum jasminoides* (J. Paxton), *Chrysanthemum* sp. (L.), *Petunia* sp. (Juss), *Lycianthes rantonnetii* (Carrière) Bitter, and *Brugmansia* sp. (Pers). Wild hosts comprise ten species, with woody and herbaceous plants equally represented (4 each) and shrubs less common (2). Key wild hosts include *Solanum dulcamara* (L.), *Malus sylvestris* (L.) Mill., *Pyrus amygdaliformis* (Vill.), *Jacobaea vulgaris* (Gaertn.), and *Portulaca oleracea* (L.).

**Figure 4:**
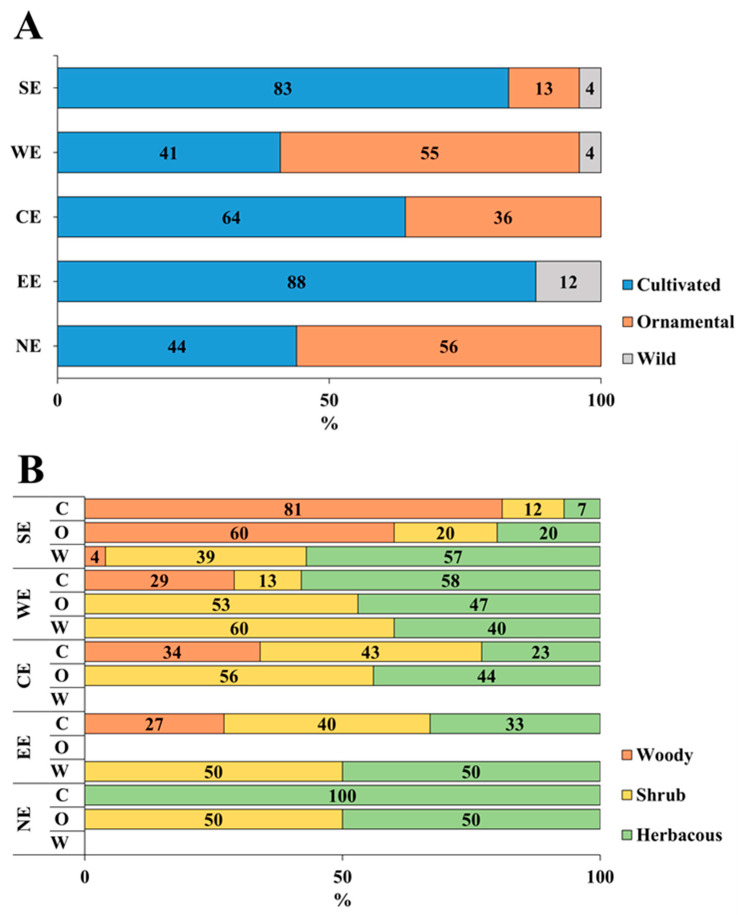
(**A**) Percentage (%) of cultivated, ornamental, and wild species, and (**B**) growth form distribution for each of these categories of reported species infected by viroids in the five European geographical regions. CE = Central Europe; EE = Eastern Europe; NE = Northern Europe; SE = Southern Europe; WE = Western Europe; C = cultivated; O = Ornamental; W = Wild.

Across Europe, clear regional contrasts emerged in the distribution of host plants (Figure 4A,B).

Overall, cultivated hosts dominate in SE, CE, and EE, while ornamental hosts are more prevalent in WE and NE. The proportion of wild hosts remained consistently low (<15%) across Europe, suggesting that most records originate from managed agricultural, urban, and greenhouse environments. Moreover, a latitudinal gradient is evident across Europe, where woody and shrubby plants are more common in the SE and CE, whereas herbaceous hosts increase toward the NE. This likely reflects both climatic constraints on woody vegetation and regional differences in land use and plant trade patterns. The key species as viroid hosts were again confirmed to be cultivated and ornamental, highlighting the significant role of human activity in the viroids’ threat [80]. However, wild species have never been deeply investigated, leaving a gap in understanding the role they play in viroid epidemiology.

## 4. Biological and Genetic Features of Viroids in the European Continent

### 4.1. Taxonomic Diversity and Dominant Species

Europe harbors a remarkably high diversity of viroids, with 32 out of the 45 viroid species formally recognized by the ICTV [40], encompassing all eight recognized genera, detected in cultivation fields, nurseries, and /or natural settings (Table 2).

**Figure 5:**
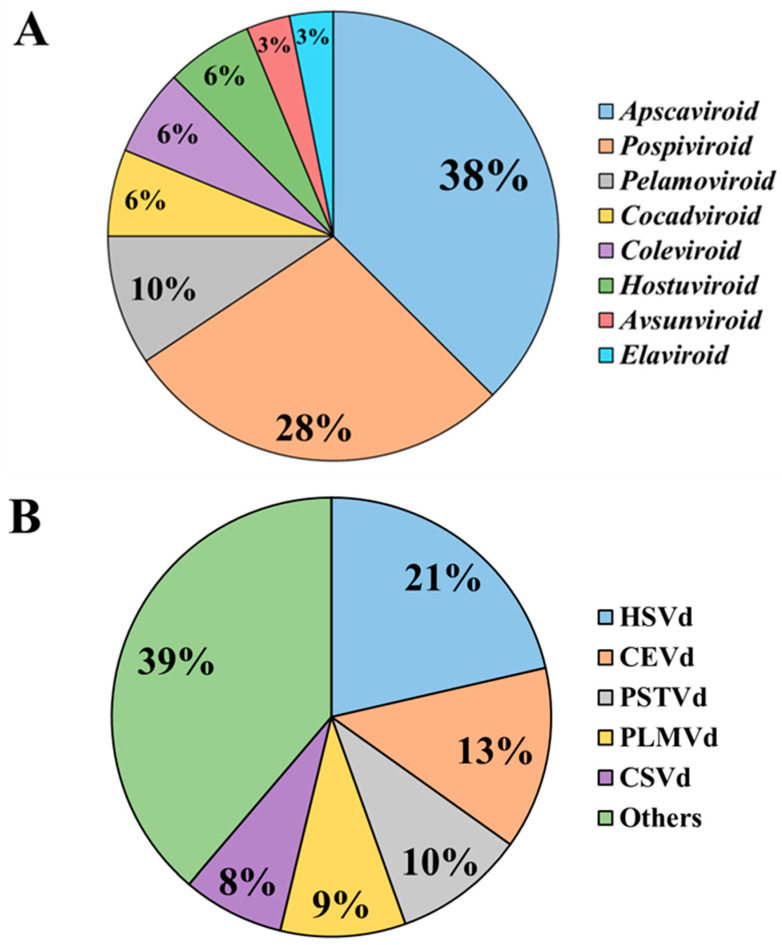
Percentage (%) of (**A**) viroid genera and (**B**) the main reported viroids, including first and successive reports, across Europe. All eight recognized genera are present, with *Apscaviroid*, *Pospiviroid*, and *Pelamoviroid* being the most represented, establishing the Pospiviroidae family as the predominant one in the European area. *Hostuviroid impedihumuli* (HSVd), *Pospiviroid exocortiscitri* (CEVd), *Pospiviroid fusituberis* (PSTVd), *Pelamoviroid latenspruni* (PLMVd), and *Pospiviroid impedichrysanthemi* (CSVd) together account for approximately 61% of all viroid reports in Europe.

The main ones are *Apscaviroid* (38%), *Pospiviroid* (28%), and *Pelamoviroid* (10%), making Pospiviroidae the predominant family in Europe. (Figure 5A). In addition, two viroid-like RNAs (i.e., GHVd and CarSV-1) were also detected. Differently, thirteen viroids have never been reported in Europe, including *Apscaviroid betadiospyri* (PVd-2), *Apscaviroid dendrobii* (DVd), *Apscaviroid diospyri* (PVd), *Apscaviroid etacitri* (CVd-VII), *Apscaviroid japanvitis* (JGVd), *Apscaviroid latenspruni* (PLVd-1), *Apscaviroid litchis* (LVd), *Cocadviroid cadangi* (CCCVd), *Cocadviroid tinangajae* (CTVd), *Coleviroid betacolei* (CbVd2), *Coleviroid epsiloncolei* (CbVd-5), *Coleviroid zetacolei* (CbVd-6), and *Pospiviroid machoplantae* (TPMVd). Their absence is likely due to the lack or limited presence of suitable hosts, as well as restricted geographical distribution in extra-European areas, such as in the case of CCCVd and LVd [81,82].

The first viroids identified in Europe in the 1970s were CSVd on *Chrysanthemum* sp. (L.) in Belgium [83] and Germany [84], although the specific host plants were not reported in the latter country. PSTVd was detected in the United Kingdom on *Solanum tuberosum* [85], HSVd in the Netherlands on *Cucumis sativus* [86], and PLMVd was detected in France in *P. persica* (L.) Batsch [87]. They were followed by CEVd in Italy on *Citrus medica* (L.), *Citrus* × *limon* (L.) Burm. f., and *Citrus* × *sinensis* (L.) Osbeck [88] (Table 2). The detection of additional viroids reflected the progressive evolution of diagnostic techniques, with certain species remaining unreported for years, while others were rapidly identified as methodologies advanced. Currently, the most frequently described viroids across the continent account 61% of reports and include HSVd (21%)*,* CEVd (13%), PSTVd (10%)*,* PLMVd (9%), and CSVd (8%), with others equal or less than 5% (Figure 5B).

### 4.2. Host Range and Ecological Specialization

The main viroids in Europe show different host ranges and ecological patterns, with HSVd and CEVd characterized by a broad spectrum and greater epidemiological risk, PSTVd mainly linked to the ornamental sector, PLMVd preferentially associated with cultivated woody species, and CSVd with natural reservoirs in wild hosts that promote its persistence and spread.

**Figure 6:**
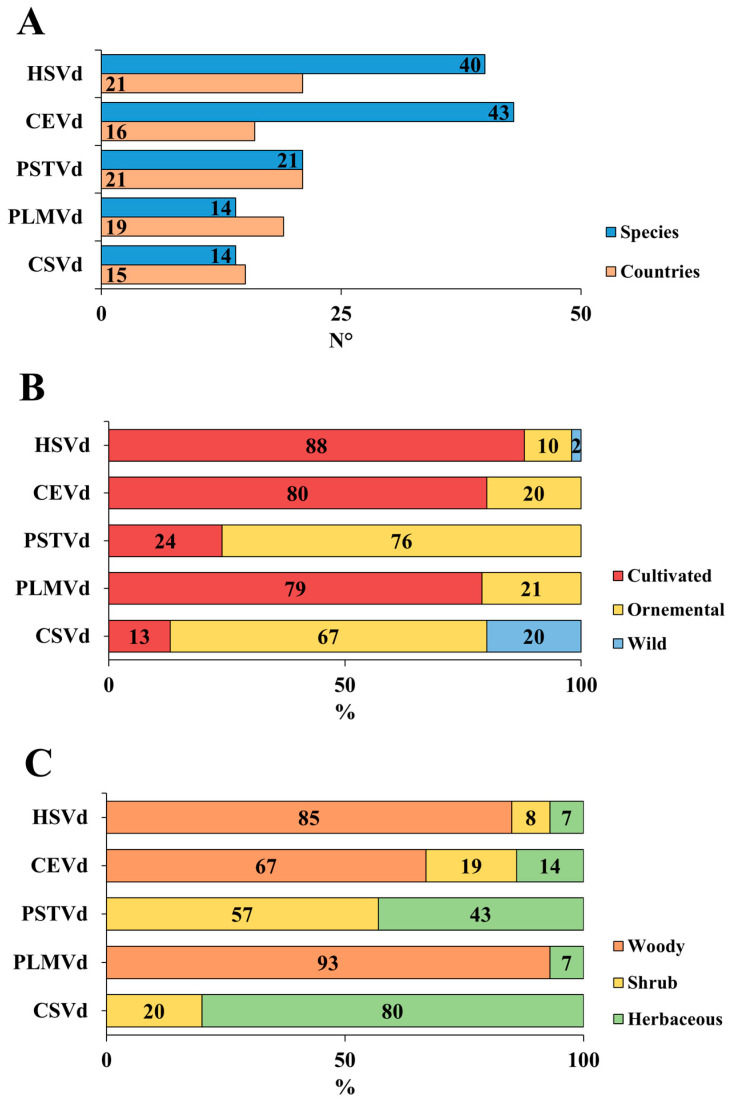
(**A**) Number of host species and number of countries, (**B**) percentages of cultivated, ornamental, and wild hosts, and (**C**) percentages of woody, shrub, and herbaceous plants associated with the five main viroids distributed across Europe. HSVd = *Hostuviroid impedihumuli*; CEVd = *Pospiviroid exocortiscitri*; PSTVd = *Pospiviroid fusituberis*; PLMVd = *Pelamoviroid latenspruni*; CSVd = *Pospiviroid impedichrysanthemi*.

HSVd was reported since 1974 and is currently present in 21 countries spanning all European geographical regions, infecting 40 plant species (Figure 6A).

CEVd, first detected in 1978, is now spread in 16 countries across all continent except the NE and infects around 43 plant species. PSTVd, identified since 1973, occurs in 21 countries across all European continent but is associated with only 21 plant species, markedly fewer than those of other viroids. PLMVd, known since 1976, has been reported in 19 countries across all macroareas, except the NE, and infects 14 plant species. Finally, CSVd has been detected in 15 countries across all Europe, affecting 14 plant species. HSVd hosts include cultivated woody plants, primarily from the *Citrus* and *Prunus* genera, as well as *Malus domestica* (Suckow) Borkh., *Mespilus germanica* (L.) Kuntze, *Pistacia vera* (L.), and *Pyrus communis* (L.) [89,90,91,92,93,94,95] (Figure 6B and 6C). Among shrubs, it was detected in *Punica granatum* (L.) and in *V. vinifera* (L.) scions and rootstocks [96,97,98]. In herbaceous plants, HSVd occurs in major crop species such as *Cucumis sativus* (L.) and *Humulus lupulus* (L.) [99,100]. Although less frequently observed in ornamental species, HSVd has been identified in woody, shrub, and herbaceous hosts, including *Morus alba* (L.), *Prunus cerasifera* (Ehrh.), *Hibiscus rosa-sinensis* (L.), and *Chrysanthemum* sp. (L.), as well as in the wild woody species *M. sylvestris* [91,99,101,102,103]. CEVd primarily infects cultivated woody plants, especially those belonging to the *Citrus* genus and *Ficus carica* (L.), followed by shrubs such as *Fortunella margaria* (Lour.) Swingle, *V. vinifera* (L.) and several major herbaceous crops, including *Brassica rapa* (L.), *Daucus carota* (L.), *S. lycopersicum* (L.) and *Solanum melongena* (L.) [49,104,105,106]. Unlike HSVd, CEVd exhibits a broader host range among ornamental shrub and herbaceous plants, including *Cestrum nocturnum* (L.), *Cestrum* sp. (L.), *H. rosa-sinensis* (L.), *L. rantonnetii* (L.), *Nematanthus* sp. (Schrad.), and *S. jasminoides* (J. Paxton), as well as *Verbena* sp. (L.), and *Verbena × hybrida* [49,68,107,108,109,110]. PSTVd presence in cultivated plants is limited to shrubs such as *Physalis peruviana* (L.) and *Solanum muricatum* (Aiton), and to herbaceous crops such as *S. lycopersicum* (L.) and *S. tuberosum* (L.) [29,44,111,112]. Notably, PSTVd infection represents the only documented case of viroid infection on *Matricaria chamomilla* (L.) in Europe [113]. In contrast, PSTVd shows a broader host range among ornamental plants, including shrubs such as *Argyranthemum frutescens* (L.), *Brugmansia sanguinea* (Ruiz & Pav.) D. Don, *Brugmansia* sp. (Pers.), *Brugmansia suaveolens* (Willd.) Sweet, *Cestrum* sp. (L.), *Datura* sp. (L.), *L. rantonnetii* (L.), *S. jasminoides* (J. Paxton), *Solanum pseudocapsicum* (L.), and *Streptosolen jamesonii* (Benth.) Miers, as well as herbaceous plants such as *Calibrachoa* sp. (Cerv.), *Chrysanthemum* sp. (L.), *Dahlia* sp. (Cav.), *Diascia* sp. (Link & Otto), and *Petunia* sp. (Juss.), including *Petunia × hybrida* [114,115,116,117,118,119,120,121,122]. PLMVd infection mainly affects cultivated woody plants, with several species belonging to the genus *Prunus*, followed by *Cydonia oblonga* (Mill.), *Diospyros kaki* (L.f.), *Juglans regia* (L.), and *P. communis* (L.) [49,95,123,124]. No natural infections were reported in shrub or herbaceous cultivated plants, nor in ornamental species, regardless of growth form. Interestingly, wild plants were also identified as hosts, including the woody species *Crataegus monogyna* (Jacq.) and *P. amygdaliformis* (Vill.), as well as the herbaceous species *Sorghum halepense* [49,123]. CSVd distribution is primarily concentrated among herbaceous hosts, particularly ornamental species, whereas in cultivated crops, detections were limited to *S. lycopersicum* (L.) and *S. tuberosum* (L.) [108,112]. Accordingly, CSVd was more frequently identified in ornamental shrubs such as *A. frutescens* (L.) and *S. jasminoides* (J. Paxton), and especially in herbaceous plants including *Calibrachoa* sp. (Cerv.), *Chrysanthemum* sp. (L.), *Chrysanthemum × grandiflorum*, *Petunia* sp. (Juss), *Petunia × hybrida*, *Vinca major* (L.), and *Vinca minor* (L.) [45,48,108,109,110,125]. While these viroids differ in their host ranges and ecological associations across Europe, the symptoms they cause are often difficult to distinguish, partly due to their latent behavior.

### 4.3. Compendium of Viroid Symptomatology

Across main European viroids (HSVd, CEVd, PSTVd, PLMVd, and CSVd), infections range from severe host-specific manifestations to latent or indistinguishable forms, underscoring both their epidemiological complexity and diagnostic challenges.

Symptoms caused by HSVd vary widely depending on the host species. In *Citrus* spp. (L.), the viroid induces stunting, leaf rolling and mottling, reduced fruit size and yield, and bark scaling or corking in specific scion-rootstock combinations. Certain variants are also associated with citrus cachexia/xyloporosis [126,127]. In *Prunus* spp. (L.), infections may remain latent, as in *P. armeniaca* (L.), or produce dapple fruit symptoms, as observed in *P. avium* (L.), *P. domestica* (L.), and *P. persica* (L.) Batsch [128,129]. In *V. vinifera* (L.), infections are frequently symptomless, although some vines show shortened, spindly canes and small leaves [130,131]. In *C. sativus* (L.), HSVd causes leaf rugosity and chlorosis, crumpled flowers, and pale or yellow bottle-shaped fruits [99]. In *H. lupulus* (L.), yellowish-green leaves and drooping petioles appear from early to mid-season, followed by stunting of both main and lateral bines; visible stunting typically develops only after several years, and cone production may be reduced [132]. In *M. alba* (L.), symptoms include vein clearing, yellow speckling, and leaf deformation [133]. Symptoms associated with CEVd can be challenging to identify, as infections may be symptomless or masked by co-infections with other viroids or pathogens [68,134,135]. In *Citrus* spp. (L.), CEVd causes exocortis disease, characterized by bark scaling, dwarfing, severe stunting, and leaf epinasty. In *F. carica* (L.), infection leads to leaf mosaic and malformations [136,137]. In *S. lycopersicum* (L.) and *S. melongena* (L.), symptoms include stunted growth and downward-curling, rough leaves [138]. Among ornamentals, *Gynura aurantiaca* (Blume) DC., the first species historically identified as a CEVd host, exhibits stunting, leaf epinasty, and midvein necrosis [139]. PSTVd infections are often latent, with only certain species or cultivars displaying visible symptoms [140]. In *S. lycopersicum* (L.), these include stunting, leaf curling, bent leaves, chlorosis, and reduced fruit size. In *S. tuberosum* (L.), infected plants grow upright with small leaflets, show reduced development, and produce distorted tubers with growth cracks and the characteristic spindle-shaped deformation [141,142]. In *Matricaria chamomilla* (L.), symptoms include pronounced stunting, shortened rosette nodes, reduced rosette size, inhibited flowering, yellowish leaves, and late-stage leaf necrosis. *Chrysanthemum* sp. (L.) develops light green young leaves, stunting, small leaves and flowers, and reduced rooting ability [113,143]. As with many viroids, many PLMVd-infected hosts remain asymptomatic or exhibit only mild symptoms [123]. However, several *Prunus* species exhibit clear and sometimes severe manifestations. In *P. persica* (L.) Batsch, these include leaf chlorotic mosaics or blotches, marginal chlorosis, and generalized yellowing, with phenotype expression linked to sequence polymorphisms. Some variants induce peach calico, characterized by extensive leaf albinism. Additional symptoms involve fruit malformations (misshapen or discolored fruit, suture cracking), enlarged or rounded stones, and bud necrosis; severe infections may delay foliation, flowering, and fruit ripening, and can reduce tree vigor and lead to premature decline [144,145,146]. In *P. domestica* (L.), the viroid causes bark necrosis and splitting on branches and trunk, and young shoots appear dwarfed due to shortened internodes [144]. Outside *Prunus*, only *Diospyros kaki* (L.f.) shows symptoms, namely fruit deformation [147]. CSVd infections are generally latent, with a notable exception in *Chrysanthemum* sp. (L.), where the viroid causes stunting, reduced flower size, petal bleaching or discoloration, leaf chlorosis or mottling, poor rooting, and altered flowering patterns [143]. Taken together, symptom variability and latent infections point to an underlying genetic complexity that can only be resolved through population-level genomic analyses.

### 4.4. Genetic Diversity, Recombination and Population Structure

European nucleotide sequences populations of HSVd, CEVd, PSTVd, PLMVd, and CSVd exhibit highly variable genetic diversity, quasispecies complexity, recombination signatures, and differentiation patterns shaped by host genus, geographic origin, and their interactions.

HSVd population displayed a guanine and cytosine (G + C) content of 56%, with an high mean similarity of 99% among sequences (82–100% range), and a low genetic diversity (π; 0.010 ± 0.003), with 181 haplotypes (Hd = 0.959 ± 0.007) and a prevalence of three of them found in 55, 27 and 17 sequences (other less or equal to five; Table 3).

In contrast, the CEVd population shows a higher G + C content of 60%, a lower mean sequence similarity of 96% (91–100%) and a higher π value (0.037 ± 0.006). Haplotypes were 54 (*Hd* = 0.985 ± 0.006), with a very slight prevalence of two haplotypes with six and five sequences (others with four or fewer). PSTVd population showed a G + C content of 58%, with a high mean similarity of 98% among sequences (92–100%) and a low π value (0.017 ± 0.004), with 134 haplotypes (Hd = 0.995 ± 0.001) with a slightly higher prevalence of two of them with seven and six sequences (other less or equal to four). PLMVd and CSVd population exhibited the same G + C content of 53% but a big difference in mean similarity among sequences with 93 (81–100%) and 98% (95–100%), respectively, as well as in π value (0.069 ± 0.007 and 0.015 ± 0.003, respectively). Moreover, PLMVd showed 232 haplotypes (Hd = 0.999 ± 0.001) and CSVd 17 (Hd = 0.9508 ± 0.025), with neither showing clear haplotype prevalence. Recombination analysis performed with RDP4 (RDP, GENECONV, BootScan, MaxChi, Chimaera, SiScan, and 3Seq algorithms), identified two HSVd isolates (EU925591, JX401927) and 34 PLMVd (EU708846-EU708848; KX430152-KX430176; MK929590; MK929592-MK929593; ON513342; Supplementary Table S4) with robust multi-method support for a recombinant origin, in agreement with earlier reports showing that, although rare, recombination contributes to viroid diversity [148,149,150]. Notably, all recombinant sequences were detected in woody species (*M. sylvestris*, *P. armeniaca* (L.) and *P. persica* (L.) Batsch), and the major and minor parental sequences involved in recombination also predominantly originated from woody hosts (*P. armeniaca* (L.) and *P. persica* (L.) Batsch), except for a single case involving *H. lupulus* (L). Moreover, most recombinant sequences were reported from Mediterranean countries (Cyprus, Greece, Italy, Spain, and Türkiye), with Hungary representing the only exception. These findings confirmed the central role of SE in shaping viroid dynamics, as observed earlier, and further support the environmental suitability of the Mediterranean basin for viroids [49]. Only HSVd and PSTVd showed significant results in Taijima’s neutrality test (D), with estimates of −2.1 (*p* = 2.8 × 10^−2^) and −2.4 (*p* = 1.8 × 10^−2^), respectively. Quasispecies behavior was confirmed in each population, characterized by numerous genetic variants capable of becoming dominant and leading to the emergence of novel infectious strains under favorable conditions [151]. However, using only European sequences for the analyses revealed specific insights, revealing distinct trends within each viroid population in Europe. PLMVd and CEVd displayed higher levels of sequence divergence and haplotype richness, with no dominant haplotypes across populations, indicating their heterogeneity and putative dynamic evolutionary nature. In contrast, HSVd and CSVd show markedly more conservative population structures, characterized by high sequence similarity, low nucleotide diversity, and a restricted set of closely related haplotypes, and, in the case of HSVd, by strong purifying selection acting on the genome. PSTVd occupies an intermediate position, with substantial haplotype diversity but moderate nucleotide divergence and evidence of purifying selection acting on its variant cloud. These trends were supported by previous studies about these viroid populations [152,153,154,155,156]. However, viroid behavior can vary drastically under different host and/or environmental conditions, due to the plasticity and adaptability conferred by quasispecies conformations and their unpredictable reaction due to host–pathogen interactions [151,157].

As a result, geographical (country; C) and taxonomic (genus; G) factors, including their interactions, were evaluated to assess their putative influence on viroid genetic variation (Table 4).

HSVd exhibited significant differentiation among genera (Φ_ST_ = 0.508; *p* = 0.001) and countries (Φ_ST_ = 0.179; *p* = 0.001), with a high level of differentiation observed in combined interactions (Φ_ST_ = 0.573; *p* = 0.001). These results indicated that host species, rather than European geographic origin, primarily contributed to population structure, confirming that host genus also plays a major role in driving variance within European viroid populations [158]. CEVd showed low differentiation among genera (Φ_ST_ = 0.115; *p* = 0.0090, not significant), but moderate to high differentiation among countries (Φ_ST_ = 0.337; *p* = 0.001) and combined interactions (Φ_ST_ = 0.492; *p* = 0.001), suggesting that geographic factors play a stronger role than host in shaping European CEVd populations. However, this appears to contrast with the broad host range and the sequence variation observed in the field, which have highlighted a host-dependent component in the genetic variability of CEVd, likely due to the limited dataset available [159,160]. PSTVd presents a high and intermediate level of differentiation among genera (Φ_ST_ = 0.465; *p* = 0.001) and countries (Φ_ST_ = 0.339; *p* = 0.001), with a high combined differentiation level (Φ_ST_ = 0.448; *p* = 0.001), suggesting both host and European geographic origin contributed substantially to its population structures as observed in previous research on PSTVd [33,120]. PLMVd showed contrasting patterns, with higher differentiation among countries (Φ_ST_ = 0.638; *p* = 0.001) than among genera (Φ_ST_ = 0.403; *p* = 0.001), reflecting a stronger European geographic influence on genetic structuring, in accordance with the previously reported tendency toward geographic clustering [161]. By contrast, the combined interactions (Φ_ST_ = 0.302) are lower, suggesting that geographic and host factors are largely independent, each with a prominent role in shaping genetic variation. This interpretation is consistent with above reported epidemiological data, which showed widespread occurrence across European countries but limited host diversity, mainly woody plants, with *P. persica* (L.) Batsch as the predominant host [49]. CSVd displayed moderate differentiation across genera (Φ_ST_ = 0.251; *p* = 0.001) and countries (Φ_ST_ = 0.354; *p* = 0.001), with a combined interaction (Φ_ST_ = 0.356; *p* = 0.002) suggesting an additive influence of host and European geography. Hosts and geography had yet to be shown to have a moderate influence on CSVd population variability, supporting the observation for the European CSVd population [156,162]. Further studies based on a larger number of nucleotide sequences are required to reconfirm or refine the abovementioned findings and to avoid potential bias arising from the limited sequence data currently available in the NCBI database. Overall, the study of nucleotide sequence populations of the five main viroids spread in Europe indicates that both host species and geographic origin contribute to shaping the genetic structure of viroid populations. However, the relative importance of each factor varies among viroid species, and other environmental factors, such as light exposure, temperature, and humidity, which were not evaluated here, can also shape and influence their population dynamics and evolutionary changes [10].

## 5. Conclusions

Viroids are classified among the most significant plant pathogens and represent the smallest known infectious agents. Despite their extreme structural simplicity, they are fully capable of replicating within host cells and causing severe diseases in a broad range of plant species worldwide, including those in Europe.

Data obtained from different sources (i.e., peer-reviewed articles, institutional documents, conference proceedings, and European and Mediterranean Plant Protection Organization international database) show that (i) the acceleration of molecular tool development, since the first identification of viroids in the early 1970s, promoted an increase in European viroid reporting, particularly during the late 1990s and 2010s, contributing to (ii) the identification of 35 countries hosting viroids, distributed into different European geographical regions. Among them, SE, under the influence of Mediterranean climate, emerged as a primary hotspot, while distinct macro-regional trends revealed (iii) the predominance of cultivated woody and shrub species in SE and CE and the increasing relevance of herbaceous and ornamental hosts moving toward NE and WE. Europe (iv) accounts for 71% of all known viroids, and the main widespread species are HSVd, CEVd, PSTVd, PLMVd, and CSVd, which exhibit markedly different ecological behaviors and epidemiological patterns. HSVd and CEVd showed exceptionally broad host spectra and high epidemiological risk, PSTVd displayed a primary association with ornamental plants, PLMVd was preferentially associated with cultivated woody species, and CSVd persisted also in wild plant reservoirs, promoting its long-term maintenance and dissemination. Moreover, (v) their biological impact is equally variable, ranging from severe host-specific symptomatology to latent infections that complicate detection and phytosanitary surveillance. At the genomic level, European viroid populations showed highly heterogeneous patterns of diversity, quasispecies dynamics, recombination, and population differentiation. The relative influence of host genus and geographic origin (i.e., country) diverged substantially among viroids, confirming that their evolution in Europe is shaped by multifactorial pressures rather than a single dominant driver. Overall, these findings highlight the need for continuous surveillance, broader sequence acquisition, and integrative ecological-genomic approaches to fully understand and manage both established and newly emerging viroid populations across Europe.

## Figures and Tables

**Table 1:** List of the 117 species reported as viroid hosts in Europe, categorized by type (cultivated, ornamental, wild plants) and ranked according to the number of newly reported viroid-host combinations and/or new geographical distribution records.

Specie	N° of Reports	Type (%)
*Vitis vinifera*	80	Cultivated (59%)
*Prunus persica*	57	
*Prunus armeniaca*	42	
*Solanum lycopersicum*	39	
*Citrus* spp.	38	
*Citrus* × *sinensis*	37	
*Citrus* × *limon*	35	
*Prunus domestica*	27	
*Malus domestica*, *Pyrus communis*	26	
*Humulus lupulus*	19	
*Citrus* × *clementina*, *Citrus* × *paradise*, *Citrus reticulata*	15	
*Solanum tuberosum*	12	
*Prunus avium*	10	
*Citrus* × *aurantifolia*	8	
*Citrus medica*, *Ficus carica*, *Prunus persica* var. *nucipersica*	6	
*Citrus* × *bergamia*, *Citrus* × *latifolia*, *Cydonia oblonga*, *Persea americana*, *Prunus salicina*, *Punica granatum*, *Solanum melongena*	5	
*Citrus* × *aurantium*, *Citrus* × *deliciosa*, *Solanum muricatum*	4	
*Citrus* × *limetta*, *Citrus* × *limonia*, *Citrus* × *limonimedica* ‘*Florentina*’, *Citrus* × *lumia*, *Citrus* × *meyeri*, *Citrus japonica*, *Cucumis sativus*, *Eriobotrya japonica*, *Physalis peruviana*, *Pistacia vera*, *Prunus dulcis*, *Vitis vinifera* (rootstock)	3	
*Brassica rapa*, *Capsicum annuum*, *Citrus* × *macrophylla*, *Daucus carota*, *Fortunella margarita*	2	
*Citrus* × *limettioides*, *Citrus* × *tangelo*, *Citrus maxima*, *Citrus reticulata × sinensis*, *Citrus* × *deliciosa* × *Citrus* × *paradisi*, *Citrus* × *grandis*, *Citrus* × *limon* × *Citrus medica*, *Citrus* × *mitis*, *Citrus* × *myrtifolia*, *Citrus* × *volkameriana*, *Diospyros kaki*, *Fortunella margarita* × *Citrus* × *clementina*, *Juglans regia*, *Matricaria chamomilla*, *Mespilus germanica*, *Prunus cerasus*, *Prunus fenzliana*, *Prunus insititia*, *Prunus tomentosa*, *Vicia faba*	1	
*Solanum jasminoides*	34	Ornamental (32%)
*Chrysanthemum* sp.	21	
*Lycianthes rantonnetii*	16	
*Brugmansia* sp.	13	
*Petunia* spp.	11	
*Argyranthemum frutescens*	9	
*Streptosolen jamesonii*	7	
*Calibrachoa* sp., *Petunia* × *hybrida*	6	
*Cestrum nocturnum*, *Coleus blumei*, *Hibiscus rosa-sinensis*, *Verbena* sp., *Vinca minor*	5	
*Cestrum* sp.	4	
*Dahlia* sp., *Plectranthus* sp.	3	
*Celosia cristata*, *Celosia plumosa*, *Chrysanthemum* × *grandiflorum*, *Datura* sp., *Portulaca* spp., *Solanum pseudocapsicum*	2	
*Brugmansia sanguinea*, *Brugmansia suaveolens*, *Brunfelsia undulata*, *Dianthus caryophyllus*, *Diascia* sp., *Iresine herbstii*, *Malus baccata*, *Morus alba*, *Nematanthus* sp., *Prunus cerasifera*, *Ptilotus macrocephalus*, *Solanum jasminoides*, *Verbena* × *hybrida*, *Vinca major*, *Vinca* sp.	1	
*Malus sylvestris*, *Solanum dulcamara*	8	Wild (9%)
*Pyrus amygdaliformis*	5	
*Jacobaea vulgaris*	4	
*Portulaca oleracea*	2	
*Crataegus monogyna*, *Crataegus* sp., *Solanum nigrum*, *Sorghum halepense*, *Vitis vinifera* subsp. *sylvestris*	1	

**Table 2:** The 32 viroid species and two viroid-like RNAs recorded in European countries between 1972 and 2025 represent all eight recognized genera. The year of the first report, the first host(s), and the country of the first report are indicated.

Specie	Other Name	Year	First Host/s	Country
*Apscaviroid alphaflavivitis*	GYSVd1	1995	*Vitis vinifera*	Germany
*Apscaviroid austravitis*	AGVd	2014	*Vitis vinifera*	Italy
*Apscaviroid betaflavivitis*	GYSVd2	2014	*Vitis vinifera*	Italy
*Apscaviroid cicatricimali*	ASSVd	1998	*Pyrus communis*	Greece
*Apscaviroid curvifoliumcitri*	CBLVd	1996	*Citrus × sinensis*	Türkiye
*Apscaviroid epsiloncitri*	CVd-V	2008	*Citrus* spp.	Spain
*Apscaviroid fossulamali*	ADFVd	1996	*Malus domestica*	Italy
*Apscaviroid latensvitis*	GLVd	2018	*Vitis vinifera*	Italy
*Apscaviroid maculamali*	ACFSVd	2019	*Malus domestica*	Austria
*Apscaviroid nanocitri*	CDVd	2000	*Citrus × meyeri*	Türkiye
*Apscaviroid pustulapyri*	PBCVd	1991	*Pyrus communis*	France
*Apscaviroid zetacitri*	CVd-VI	2024	*Hibiscus rosa-sinensis*	Italy
*Avsunviroid albamaculaperseae*	ASBVd	1987	*Persea americana*	Spain
*Cocadviroid latenshumuli*	HLVd	1988	*Humulus lupulus*, *Vitis vinifera*	Germany
*Cocadviroid rimocitri*	CBCVd	1988	*Citrus medica*	Spain
*Coleviroid alphacolei*	CbVd-1	1990	*Coleus blumei*	Germany
*Coleviroid gammacolei*	CbVd-3	1996	*Coleus blumei*	Germany
*Elaviroid latensmelongenae*	ELVd	2024	*Solanum melongena*	Spain
*Hostuviroid impedihumuli*	HSVd	1974	*Cucumis sativus*	Netherlands
*Hostuviroid latensdahliae*	DLVd	2013	*Dahlia* sp.	Netherlands
*Pelamoviroid latenspruni*	PLMVd	1976	*Prunus persica*	France
*Pelamoviroid maculachrysanthemi*	CChMVd	2024	*Chrysantemum* sp.	France
*Pelamoviroid malleusmali*	AHVd	2019	*Malus domestica*	Italy
*Pospiviroid alphairesinis*	IVd-1	1996	*Irsine herbstii*	Germany
*Pospiviroid apicimpeditum*	TASVd	1996	*Solanum pseudocapsicum*	Germany
*Pospiviroid chloronani*	TCDVd	2004	*Solanum lycopersicum*	Belgium
*Pospiviroid exocortiscitri*	CEVd	1978	*Citrus* spp.	Italy
*Pospiviroid fusituberis*	PSTVd	1973	*Solanum tuberosum*	United Kingdom
*Pospiviroid impedichrysanthemi*	CSVd	1972	*Chrysantemum* sp.	Belgium
*Pospiviroid latenscolumneae*	CLVd	1996	*Brunfelsia undulata*	Germany
*Pospiviroid latensportulacae*	PoLVd	2015	*Portulaca* sp.	Netherlands
*Pospiviroid parvicapsici*	PCFVd	2009	*Capsicum annuum*	Netherlands
Carnation small viroid-like RNA 1	CarSV-1	2023	*Dianthus caryophyllus*	Netherlands
Grapevine hammerhead viroid-like RNA	GHVd	2012	*Vitis vinifera*	Italy

**Table 3:** Analyses of nucleotide sequence populations, including number of sequences (N°), guanine + cytosine (G + C) content (%), similarity mean (%), similarity range (%), genetic diversity (π), number of haplotype (N°), haplotype diversity (Hd), and Tajima’s neutrality test (D), for the five main viroids reported in Europe, retrieved from the NCBI database. HSVd = *Hostuviroid impedihumuli*; CEVd = *Pospiviroid exocortiscitri*; PSTVd = *Pospiviroid fusituberis*; PLMVd = *Pelamoviroid latenspruni*; CSVd = *Pospiviroid impedichrysanthemi*; * = significant result, S.D. = ± standard deviation; S.E. = ± standard error.

Viroid	Sequences	G + C	Similarity Mean	Similarity Range	Genetic Diversity		Haplotype	Haplotype Diversity		Taijima’ s Neutrality Test	
	N°	%	%	%	π	S.D.	N°	Hd	S.E.	D	*p* Value
HSVd	315	56	99	82–100	0.010	0.003	181	0.959	0.007	−2.1	0.028 *
CEVd	71	60	96	91–100	0.037	0.006	54	0.985	0.006	−1.3	0.892
PSTVd	157	58	98	92–100	0.017	0.004	134	0.995	0.001	−2.3	0.018 *
PLMVd	253	53	93	81–100	0.069	0.007	232	0.999	0.001	−0.5	0.644
CSVd	26	53	98	95–100	0.015	0.003	17	0.9508	0.025	−1.5	0.117

**Table 4:** AMOVA results for the main European viroid nucleotide sequence populations based on sequences available in the NCBI database, considering geographical (Country; C) and taxonomical (genera; G) metadata, as well as their interactions (*G* × *C*). HSVd = *Hostuviroid impedihumuli*; CEVd = *Pospiviroid exocortiscitri*; PSTVd = *Pospiviroid fusituberis*; PLMVd = *Pelamoviroid latenspruni*; CSVd = *Pospiviroid impedichrysanthemi*; Φ_ST_ = Phi- statistic; * = significant result.

Viroid	Factor/s	Φ_ST_	*p*
HSVd	*C*	0.179	0.001 *
	*G*	0.508	0.001 *
	*G × C*	0.573	0.001 *
CEVd	*C*	0.337	0.001 *
	*G*	0.115	0.090
	*G × C*	0.492	0.001 *
PSTVd	*C*	0.339	0.001 *
	*G*	0.465	0.001 *
	*G × C*	0.448	0.001 *
PLMVd	*C*	0.638	0.001 *
	*G*	0.403	0.001 *
	*G × C*	0.302	0.001 *
CSVd	*C*	0.354	0.001 *
	*G*	0.251	0.001 *
	*G × C*	0.356	0.002 *

## Data Availability

No new data were created or analyzed in this study.

## Supplementary Materials

The following supporting information can be downloaded at: https://www.mdpi.com/article/10.3390/v18030325/s1, Supplementary Material S1. Database; Supplementary Table S1. List of 51 European countries, including geographically European sovereign states and transcontinental countries with partial European territories considered in this study; Supplementary Table S2. Accession numbers of nucleotide sequences of the five main viroids reported in Europe. HSVd = *Hostuviroid impedihumuli*; CEVd = *Pospiviroid exocortiscitri*; PSTVd = *Pospiviroid fusituberis*; PLMVd = *Pelamoviroid latenspruni*; CSVd = *Pospiviroid impedichrysanthemi;* Supplementary Table S3. Ranking of countries in Europe based on the number of viroid reports recovered in this study; Supplementary Table S4. Recombinant analysis results obtained from European nucleotide sequence populations of *Hostuviroid impedihumuli* (HSVd) and *Pelamoviroid latenspruni* (PLMVd) retrieved from the NCBI database. MC p = Multiple comparison correction *p*-value; bb = beginning breakpoint (bp); eb = ending breakpoint (bp); p-values for Recombination Detection Program (RDP), Bootscan, MaxChi, Chimaera, SiScan, and 3Seq algorithms; map = major parent; %_map_ = percentage of similarity between the major parent and the recombinant sequence; %_mip_ = percentage of similarity between the minor parent and the recombinant sequence; --- = no significant result; AJ297832 = *Prunus armeniaca* (L.), Cyrprus; ON513443 = *Prunus persica* (L.) Batsch, Italy; OQ366357 = *Humulus lupulus* (L.), Türkiye. Only significant results were reported (*p* < 0.05).

## Abbreviations

ACFSVd	*Apscaviroid maculamali*
ADFVd	*Apscaviroid fossulamali*
AGVd	*Apscaviroid austravitis*
AHVd	*Pelamoviroid malleusmali*
AMOVA	Analysis of Molecular Variance
ASBVd	*Avsunviroid albamaculaperseae*
ASSVd	*Apscaviroid cicatricimali*
CarSV-1	Carnation small viroid-like RNA 1
CBCVd	*Cocadviroid rimocitri*
CBLVd	*Apscaviroid curvifoliumcitri*
CbVd-1	*Coleviroid alphacolei*
CbVd-3	*Coleviroid gammacolei*
CChMVd	*Pelamoviroid maculachrysanthemi*
CDVd	*Apscaviroid nanocitri*
CE	Central Europe
CEVd	*Pospiviroid exocortiscitri*
CLVd	*Pospiviroid latenscolumneae*
CSVd	*Pospiviroid impedichrysanthemi*
CVd-V	*Apscaviroid epsiloncitri*
CVd-VI	*Apscaviroid zetacitri*
DLVd	*Hostuviroid latensdahliae*
EE	Eastern Europe
ELVd	*Elaviroid latensmelongenae*
EPPO	European and Mediterranean plant protection organization
GHVd	Grapevine hammerhead viroid-like RNA
GLVd	*Apscaviroid latensvitis*
GYSVd1	*Apscaviroid alphaflavivitis*
GYSVd2	*Apscaviroid betaflavivitis*
HLVd	*Cocadviroid latenshumuli*
HSVd	*Hostuviroid impedihumuli*
HTS	High-throughput sequencing
IVd-1	*Pospiviroid alphairesinis*
ICTV	International Committee on Taxonomy of Viruses
NCBI	National center for biotechnology information
NE	Northern Europe
NGS	Next-generation sequencing
PAGE	Polyacrylamide gel electrophoresis
PBCVd	*Apscaviroid pustulapyri*
PCFVd	*Pospiviroid parvicapsici*
PLMVd	*Pelamoviroid latenspruni*
PoLVd	*Pospiviroid latensportulacae*
PSTVd	*Pospiviroid fusituberis*
RNQP	Regulated non-quarantine pests
RT-LAMP	Reverse transcription loop-mediated isothermal amplification
RT-PCR	Reverse transcription polymerase chain reaction
SE	Southern Europe
TASVd	*Pospiviroid apicimpeditum*
TCDVd	*Pospiviroid chloronani*
WE	Western Europe
