# Supplementary material for: Inside the European Plant Viroid Scenario: Continental Distribution, Host Range, and Genetic Features of the Main Viroid Populations
Source: Viruses. 2026 Mar 5;18(3):325. doi: 10.3390/v18030325 (PMC13030037; doi:10.3390/v18030325)
Supplement: Supplementary file 1 [file viruses-18-00325-s001.zip › 2026_Pedrellietal_viruses-4155259_Supplementary Table S1.pdf]

Supplementary Table S1. List of 51 European countries, including geographically European sovereign states and transcontinental countries with partial European territories considered in this study.

**Country**
Albania
Andorra
Armenia
Austria
Azerbaijan
Belarus
Belgium
Bosnia and Herzegovina
Bulgaria
Croatia
Cyprus
Czechia
Denmark
Estonia
Finland
France
Georgia
Germany
Greece
Hungary
Iceland
Ireland
Italy
Kazakhstan
Kosovo
Latvia
Liechtenstein
Lithuania
Luxembourg
Malta
Moldova
Monaco
Montenegro
Netherlands
North Macedonia
Norway
Poland
Portugal
Romania
Russia
San Marino

Serbia
Slovakia
Slovenia
Spain
Sweden
Switzerland
Türkiye
Ukraine
United Kingdom
Vatican City
