# Supplementary material for: Inside the European Plant Viroid Scenario: Continental Distribution, Host Range, and Genetic Features of the Main Viroid Populations
Source: Viruses. 2026 Mar 5;18(3):325. doi: 10.3390/v18030325 (PMC13030037; doi:10.3390/v18030325)
Supplement: Supplementary file 1 [file viruses-18-00325-s001.zip › 2026_Pedrellietal_viruses-4155259_Supplementary Table S2.pdf]

Supplementary Table S2. Accession numbers of nucleotide sequences of the five main viroids reported in Europe. HSVd= *Hostuviroid impedihumuli*; CEVd= *Pospiviroid exocortiscitri*; PSTVd= *Pospiviroid fusituberis*; PLMVd= *Pelamoviroid latenspruni*; CSVd= *Pospiviroid impedichrysanthemi.*

CEVd	CSVd	HSVd	PLMVd	PSTVd
AM774356.1	JF938538.1	AJ297830.1	AF339739.1	EF459697.1
AM774357.1	JQ685734.1	AJ297831.1	AJ550545.1	EF459698.1
AM920649.1	JQ685735.1	AJ297832.1	AJ550898.1	EF459699.1
EU094207.1	JQ685736.1	AJ297833.1	AJ550899.1	EF459700.1
EU094208.1	JQ685737.1	AJ297834.1	AJ550900.1	EF459701.1
EU447280.1	JQ685738.1	AJ297835.1	AJ550901.1	EU257478.1
EU872276.1	JQ685739.1	AJ297836.1	AJ550902.1	EU879913.1
GQ246191.1	JQ685740.1	AJ297837.1	AJ550903.1	EU879914.1
GQ246192.1	JQ685741.1	AJ297838.1	AJ550904.1	EU879915.1
GQ246194.1	JQ685742.1	AJ297839.1	AJ550905.1	EU879916.1
GU300810.1	LC430901.1	AJ297840.1	AJ550906.1	EU879917.1
HF954916.1	LC523679.1	EF151290.1	AJ550907.1	EU879918.1
HF954917.1	LC523680.1	EF151291.1	AJ550908.1	EU879919.1
HF954918.1	LC523681.1	EF523825.1	AJ550909.1	EU879920.1
HF954919.1	LC523682.1	EF523826.1	AJ550910.1	EU879921.1
HF954920.1	LC523683.1	EF523827.1	AJ550911.1	EU879922.1
HQ423166.1	LC523684.1	EF523828.1	AJ550912.1	EU879923.1
HQ452417.1	LC523685.1	EF523829.1	DQ222043.1	EU879924.1
HQ667138.1	LC523686.1	EU872277.1	DQ222044.1	EU879925.1
JN872140.1	LC523687.1	EU925588.2	DQ222045.1	EU879926.1
JQ083645.1	LC523688.1	EU925589.2	DQ222046.1	FJ872823.1
JQ083646.1	LC523689.1	EU925590.2	DQ222047.1	FR851463.1
JQ083647.1	MW013136.1	EU925591.1	DQ222048.1	GQ853457.1
JX259392.1	OK340214.1	FJ974075.2	DQ222049.1	GQ853458.1
JX259393.1	OK340215.1	FJ974076.2	DQ222050.1	GQ853459.1
JX259394.1	PP256250.1	FJ974077.2	DQ222051.1	GQ853460.1
JX259395.1		FJ974078.2	DQ222052.1	GQ853461.1
JX259396.1		FJ974079.1	DQ222053.1	GQ853462.1
JX885864.1		FJ974080.2	DQ222054.1	GQ853463.1
JX885865.1		FJ974081.2	DQ222055.1	GQ853464.1
JX885866.1		FR865478.1	DQ222056.1	GQ853465.1
JX885867.1		FR865479.1	DQ222057.1	GQ853466.1
KF265340.1		FR865480.1	DQ222058.1	GQ853467.1
KX156930.1		GQ246198.1	DQ222059.1	GQ853468.1
KX156931.1		GQ246199.1	DQ222060.1	GQ853469.1
KX156932.1		GQ246200.1	DQ222061.1	GU481090.1
KX156933.1		GQ246201.1	DQ222062.1	GU481091.1
KX819231.1		GQ249348.2	DQ222063.1	GU481092.1
KX819232.1		GQ254645.1	DQ222064.1	HQ452399.1
KX819233.1		GQ995464.1	DQ222065.1	HQ452400.1

KX819234.1	GQ995465.1	DQ222066.1	HQ452401.1
KX819235.1	GQ995466.1	DQ222067.1	HQ452402.1
KX819236.1	HE575344.1	DQ222068.1	HQ452403.1
KX819237.1	HE575345.1	DQ222069.1	HQ452404.1
MN136643.1	HE575346.1	DQ222070.1	HQ452405.1
MW013137.1	HE575347.1	DQ222071.1	HQ452406.1
MW013138.1	HF954921.1	DQ222072.1	HQ452407.1
MZ995262.1	HF954922.1	DQ222073.1	HQ452408.1
OP947755.1	HF954923.1	DQ222074.1	HQ452409.1
OP947756.1	HF954924.1	DQ222075.1	HQ452410.1
OP947757.1	HF954925.1	DQ222076.1	HQ452411.1
OP947758.1	HG518673.1	DQ222077.1	HQ452412.1
OQ127291.1	HG518674.1	DQ222078.1	HQ452413.1
OQ366358.1	HG518675.1	DQ222079.1	HQ452414.1
OQ366359.1	JX259397.1	DQ222080.1	HQ452415.1
OQ366360.1	JX259398.1	DQ222081.1	HQ452416.1
OQ366361.1	JX259399.1	DQ222082.1	HQ454914.1
OQ632305.1	JX259400.1	DQ222083.1	HQ454915.1
OR344750.1	JX259401.1	DQ222084.1	HQ454916.1
OR344751.1	JX259402.1	DQ222085.1	HQ454917.1
OR344752.1	JX259403.1	DQ222086.1	HQ454918.1
OR344753.1	JX259404.1	DQ222087.1	HQ454919.1
PP405017.1	JX259405.1	DQ222088.1	HQ454920.1
PP405018.1	JX259406.1	DQ222089.1	HQ454921.1
PP446493.1	JX259407.1	DQ222090.1	HQ454922.1
PP856225.1	JX259408.1	DQ222091.1	HQ454923.1
PP942536.1	JX259409.1	DQ222092.1	HQ454924.1
PV339782.1	JX259410.1	DQ222093.1	HQ454925.1
PV339783.1	JX259411.1	DQ222094.1	HQ454926.1
PV339784.1	JX259412.1	DQ222095.1	HQ454927.1
PV339785.1	JX259413.1	DQ222096.1	HQ454928.1
	JX259414.1	DQ222097.1	HQ454929.1
	JX259415.1	DQ222098.1	HQ454930.1
	JX259416.1	DQ222099.1	HQ454931.1
	JX259417.1	DQ222100.1	HQ454932.1
	JX401927.1	DQ222101.1	HQ454933.1
	JX430797.1	DQ222102.1	HQ454934.1
	JX430800.1	DQ222103.1	HQ454935.1
	KC137256.1	DQ222104.1	HQ454936.1
	KC137257.1	EF151292.1	HQ454937.1
	KC137258.1	EF151293.1	HQ456944.1
	KC137259.1	EF151294.1	JN559763.1
	KC137260.1	EF151295.1	JN559764.1
	KC137261.1	EF151296.1	KF419168.1
	KC137262.1	EF151297.1	KF493732.1
	KC137263.1	EF151298.1	KM204317.1
	KC137264.1	EF151299.1	KM204318.1
	KC137265.1	EF151300.1	KM204319.1
	KC137266.1	EU708818.1	KT987925.1

KC584010.1	EU708819.1	KX370618.1
KC584011.1	EU708820.1	KY936873.1
KC584012.1	EU708822.1	KY936874.1
KC584013.1	EU708823.1	KY936875.1
KC584014.1	EU708824.1	KY936876.1
KC584016.1	EU708825.1	KY936877.1
KC584017.1	EU708826.1	KY936878.1
KC584021.1	EU708827.1	KY936879.1
KC584022.1	EU708828.1	KY936880.1
KC677729.1	EU708829.1	KY936881.1
KC677730.1	EU708830.1	KY936882.1
KC878438.1	EU708831.1	KY936883.1
KC878439.1	EU708832.1	KY936884.1
KC878440.1	EU708833.1	KY936885.1
KF060659.1	EU708834.1	LC523658.1
KF060660.1	EU708835.1	LC523659.1
KF060661.1	EU708836.1	LC523660.1
KF534763.1	EU708837.1	LC523661.1
KF534764.1	EU708838.1	LC523662.1
KF534765.1	EU708839.1	LC523663.1
KJ466327.1	EU708840.1	LC523664.1
KJ466328.1	EU708841.1	LC523665.1
KJ466329.1	EU708842.1	LC523666.1
KJ466330.1	EU708843.1	LC523667.1
KJ466331.1	EU708844.1	LC523668.1
KJ466332.1	EU708845.1	LC523669.1
KJ466333.1	EU708846.1	LC523670.1
KM875543.1	EU708847.1	LC523671.1
KX156934.1	EU708848.1	LC523672.1
KX156935.1	GQ872122.1	LC523673.1
KX156936.1	GQ872123.1	LC523674.1
KX819238.1	GQ872124.1	LC523675.1
KX819239.1	GQ872125.1	LC523676.1
KX819240.1	GQ872126.1	LC523677.1
KX819241.1	GQ872127.1	LC523678.1
KX819242.1	GQ872128.1	LC654168.1
KX819243.1	GQ872129.1	LC654169.1
KX819244.1	GQ872130.1	LC654170.1
MF497538.1	GQ872131.1	LC654171.1
MF543122.1	GQ872132.1	MF140246.1
MK955873.1	GQ872133.1	MF140247.1
MK955874.1	GQ872134.1	MF140248.1
MN136644.1	GQ872135.1	MF140249.1
MN548397.1	GQ872136.1	MF140250.1
MZ196517.1	GQ872137.1	MF140251.1
MZ825532.1	GQ872138.1	MF140252.1
MZ825533.1	GQ872139.1	MF140253.1
MZ825540.1	GQ872140.1	MF140254.1
MZ825542.1	GQ872141.1	MG450357.1

MZ825544.1	GQ872142.1	MG450358.1
MZ825549.1	GQ872143.1	MK948524.1
MZ995257.1	GQ872144.1	OM867875.1
MZ995258.1	GQ872145.1	ON478345.1
MZ995260.1	GQ872146.1	ON478346.1
OL589432.1	GQ872147.1	ON478347.1
OL799308.1	GQ872148.1	ON936749.1
ON669206.1	GQ872149.1	ON936750.1
ON669207.1	GQ872150.1	ON936751.1
ON669208.1	GQ872151.1	ON936761.1
ON669209.1	JF416648.1	ON936777.1
ON669210.1	JF416649.1	ON936780.1
ON669211.1	JF927892.1	ON936781.1
ON669212.1	JF927893.1	ON936782.1
ON669213.1	JF927894.1	PP256245.1
ON669214.1	JF927895.1	PQ072294.1
ON669215.1	JF927896.1	PQ519571.1
ON669216.1	JF927897.1	PQ519572.1
ON669217.1	JF927898.1	PQ519573.1
ON669218.1	KU048778.1	
ON669219.1	KU048779.1	
ON669220.1	KU048780.1	
ON669221.1	KU048781.1	
ON669222.1	KU048782.1	
ON669223.1	KU048783.1	
ON669224.1	KU048784.1	
ON669225.1	KU048785.1	
ON669226.1	KU048786.1	
ON669227.1	KU048787.1	
ON669228.1	KU048788.1	
ON669229.1	KU048789.1	
ON669230.1	KU048790.1	
ON669231.1	KU048791.1	
ON669232.1	KU048792.1	
ON669233.1	KU048793.1	
ON669234.1	KU048794.1	
ON669235.1	KU048795.1	
ON669236.1	KU048796.1	
ON669237.1	KU048797.1	
ON669238.1	KU048798.1	
ON669239.1	KX430152.1	
ON669240.1	KX430153.1	
ON669241.1	KX430154.1	
ON669242.1	KX430155.1	
ON669243.1	KX430156.1	
ON669244.1	KX430157.1	
ON669245.1	KX430158.1	
ON669246.1	KX430159.1	
ON669247.1	KX430160.1	

ON669248.1	KX430161.1
OP885209.1	KX430162.1
OP885280.1	KX430163.1
OP885281.1	KX430164.1
OP885282.1	KX430165.1
OP885283.1	KX430166.1
OP885284.1	KX430167.1
OP885285.1	KX430168.1
OP885286.1	KX430169.1
OP885287.1	KX430170.1
OP885288.1	KX430171.1
OP885289.1	KX430172.1
OP885290.1	KX430173.1
OP885291.1	KX430174.1
OP885292.1	KX430175.1
OP885293.1	KX430176.1
OP885294.1	KY810773.1
OP885295.1	MK929586.1
OP885296.1	MK929587.1
OP885297.1	MK929588.1
OP885298.1	MK929589.1
OP885299.1	MK929590.1
OP885300.1	MK929591.1
OP885301.1	MK929592.1
OP885302.1	MK929593.1
OP885303.1	MW201797.2
OP885304.1	MW928673.1
OP885305.1	MW928674.1
OP885306.1	MW928675.1
OP885307.1	MW928676.1
OP885308.1	MW928677.1
OP885309.1	MW928678.1
OP885310.1	MW928679.1
OP885311.1	MW928680.1
OP885312.1	MW928681.1
OP885313.1	MW928682.1
OP885314.1	MW928683.1
OP885315.1	MZ289068.1
OP885316.1	MZ289069.1
OP885317.1	MZ289070.1
OP885318.1	MZ289071.1
OP885319.1	MZ289072.1
OP885320.1	MZ289073.1
OP885321.1	MZ289074.1
OP885322.1	OL311697.1
OP885323.1	OL311698.1
OP885324.1	ON428191.1
OP885325.1	ON428192.1
OP885326.1	ON513442.1

OP885327.1	ON513443.1
OP918897.1	ON513444.1
OP918898.1	ON513445.1
OP918899.1	ON513446.1
OP918900.1	PP079189.1
OP918901.1	PP079190.1
OP918902.1	PP079191.1
OP918903.1	PP079192.1
OP918904.1	PP079193.1
OP918905.1	PP079194.1
OP918906.1	PP079195.1
OP918907.1	PV254009.1
OP918908.1	PV254010.1
OP918909.1	PV254011.1
OP918910.1	PV254012.1
OP918911.1	PV254013.1
OP918912.1	PV254014.1
OP918913.1	
OP918914.1	
OP947761.1	
OQ366357.1	
OQ366365.1	
OQ366366.1	
OQ366367.1	
OQ366368.1	
OQ366369.1	
OQ366370.1	
OQ366371.1	
OR257530.1	
OR257531.1	
OR344754.1	
OR344756.1	
OR344757.1	
OR892425.1	
OR892426.1	
OR892428.1	
OR892429.1	
OR892430.1	
OR892431.1	
OR892432.1	
OR892434.1	
OR892435.1	
OR892437.1	
OR892439.1	
OR892440.1	
OR892442.1	
OR892444.1	
OR892445.1	
OR892447.1	

OR892448.1
PP079203.1
PP079204.1
PP079205.1
PP079206.1
PP079207.1
PP079208.1
PP079209.1
PQ558645.1
PQ558646.1
PQ558647.1
PQ558648.1
PQ558650.1
PQ558651.1
PQ558652.1
PQ558653.1
PQ558654.1
PQ736855.1
PQ736856.1
PQ736857.1
PQ736858.1
PQ736859.1
PQ736860.1
PQ736861.1
PQ736862.1
PQ736863.1
PQ736864.1
PQ736865.1
PQ736866.1
PV870369.1
