# Supplementary material for: Inside the European Plant Viroid Scenario: Continental Distribution, Host Range, and Genetic Features of the Main Viroid Populations
Source: Viruses. 2026 Mar 5;18(3):325. doi: 10.3390/v18030325 (PMC13030037; doi:10.3390/v18030325)
Supplement: Supplementary file 1 [file viruses-18-00325-s001.zip › 2026_Pedrellietal_viruses-4155259_Supplementary Table S3.pdf]

Supplementary Table S3. Ranking of countries in Europe based on the number of viroid reports recovered in this study.

Country	N° of reports
Italy	218
Greece	134
Türkiye	70
Spain	61
France	46
Netherlands	44
Czechia	37
Germany	29
Slovenia	29
Croatia	28
Cyprus	24
Belgium	21
Albania	18
Poland	15
Russia	13
Finland	11
Bosnia and Herzegovina	11
Montenegro	9
United Kingdom	9
Hungary	5
Serbia	4
Austria	4
Slovakia	3
Azerbaijan	3
Portugal	3
Kosovo	3
Ukraine	2
Romania	2
Switzerland	1
Bulgaria	1
Norway	1
Belarus	1
Georgia	1
Malta	1
Sweden	1
