# Supplementary material for: Inside the European Plant Viroid Scenario: Continental Distribution, Host Range, and Genetic Features of the Main Viroid Populations
Source: Viruses. 2026 Mar 5;18(3):325. doi: 10.3390/v18030325 (PMC13030037; doi:10.3390/v18030325)
Supplement: Supplementary file 1 [file viruses-18-00325-s001.zip › 2026_Pedrellietal_viruses-4155259_Supplementary Table S4.pdf]

Supplementary Table S4. Recombinant analysis results obtained from European nucleotide sequence populations of *Hostuviroid impedihumuli* (HSVd) and *Pelamoviroid latenspruni* (PLMVd) retrieved from the NCBI database. MC p = Multiple comparison correction *p*-value; bb = beginning breakpoint (bp); eb = ending breakpoint (bp); p-values for Recombination Detection Program (RDP), Bootscan, MaxChi, Chimaera, SiScan, and 3Seq algorithms; map = major parent; $\%_{map}$= percentage of similarity between the major parent and the recombinant sequence; $\%_{mip}$ = percentage of similarity between the minor parent and the recombinant sequence; --- = no significant result; AJ297832 = *Prunus armeniaca* (L.), Cyrprus; ON513443 = *Prunus persica* (L.) Batsch, Italy; OQ366357 = *Humulus lupulus* (L.), Türkiye. Only significant results were reported ($p < 0.05$).

Viroid	Isolate	Host	Country	MC p	bb	eb	RDP	Bootscan	MaxChi	Chimaera	SiScan	3Seq	map	$\%_{map}$	mip	$\%_{mip}$
HSVd	EU925591	*Prunus armeniaca*	Greece	4.56E-07	83	179	2.77E-02	1.590E-2	2.44E-02	---	4.56E-07	2.78E-02	OQ366357	99	Unknown	---
	JX401927	*Malus sylvestris*	Greece	2.95E-07	56	172	2.95E-03	2.797E-2	1.63E-04	2.41E-03	2.95E-07	---	AJ297832	97	Unknown	---
PLMVd	EU708846			2.05E-06												90
	EU708847	*Prunus persica*	Türkiye	8.20E-04	333	141	---	---	3.80E-05	1.55E-02	1.39E-03	1.48E-03	Unknown	---	ON513443	89
	EU708848			8.20E-04												89
	KX430152			5.18E-06	333	135										88
	KX430153			5.11E-06	333	135										86
	KX430154			7.87E-07	333	135										86
	KX430155	*Unknown*	Spain	1.03E-04	333	121	---	---	3.80E-05	1.55E-02	1.39E-03	1.48E-03	Unknown	---	ON513443	88
	KX430156			2.20E-03	333	135										88
	KX430157			3.32E-06	333	135										88
	KX430158			6.65E-07	333	136										88
	KX430159			3.32E-06	333	136										88

KX430160			5.11E-06	333	135										88
KX430161			3.27E-06	316	135										86
KX430162			2.43E-02	332	135										86
KX430163			3.27E-06	332	135										85
KX430164			3.22E-06	332	135										85
KX430165			3.27E-06	332	135										85
KX430166			1.41E-02	330	136										84
KX430167			3.32E-06	333	135										84
KX430168			5.57E-07	333	136										88
KX430169			2.05E-06	332	135										88
KX430170			1.51E-03	9	126										87
KX430171			6.65E-07	333	135										88
KX430172			2.05E-06	332	135										88
KX430173			3.27E-06	333	135										87
KX430174			5.11E-06	332	135										87
KX430175			2.05E-06	332	135										88
KX430176			6.65E-07	333	135										88
MK929590			4.38E-06	334	141										84
MK929592	*Prunus persica*	Hungary	3.36E-06	333	141	---	---	3.80E-05	1.55E-02	1.39E-03	1.48E-03	Unknown	---	ON513443	88
MK929593			6.28E-05	334	141										89
ON513442	*Prunus persica*	Italy	2.71E-06	331	135	---	---	3.80E-05	1.55E-02	1.39E-03	1.48E-03	Unknown	---	ON513443	83
